# Anaphylatoxins Activate Ca^2+^, Akt/PI3-Kinase, and FOXO1/FoxP3 in the Retinal Pigment Epithelium

**DOI:** 10.3389/fimmu.2017.00703

**Published:** 2017-06-15

**Authors:** Catharina Busch, Balasubramaniam Annamalai, Khava Abdusalamova, Nadine Reichhart, Christian Huber, Yuchen Lin, Emeraldo A. H. Jo, Peter F. Zipfel, Christine Skerka, Gerhild Wildner, Maria Diedrichs-Möhring, Bärbel Rohrer, Olaf Strauß

**Affiliations:** ^1^Department of Ophthalmology, Charité University Medicine Berlin, Berlin, Germany; ^2^Berlin Institute of Health, Berlin, Germany; ^3^Department of Ophthalmology, Medical University of South Carolina, Charleston, SC, United States; ^4^Department of Ophthalmology, University of Heidelberg, Heidelberg, Germany; ^5^Department of Infection Biology, Leibniz Institute for Natural Product Research and Infection Biology, Jena, Germany; ^6^Department of Ophthalmology, Section of Immunobiology, Clinic of the LMU Munich, Munich, Germany; ^7^Ralph H. Johnson VA Medical Center, Division of Research, Charleston, SC, United States

**Keywords:** anaphylatoxins, calcium signaling, FOXO1, FoxP3, retinal pigment epithelium

## Abstract

**Purpose:**

The retinal pigment epithelium (RPE) is a main target for complement activation in age-related macular degeneration (AMD). The anaphylatoxins C3a and C5a have been thought to mostly play a role as chemoattractants for macrophages and immune cells; here, we explore whether they trigger RPE alterations. Specifically, we investigated the RPE as a potential immunoregulatory gate, allowing for active changes in the RPE microenvironment in response to complement.

**Design:**

*In vitro* and *in vivo* analysis of signaling pathways.

**Methods:**

Individual activities of and interaction between the two anaphylatoxin receptors were tested in cultured RPE cells by fluorescence microscopy, western blot, and immunohistochemistry.

**Main outcome measures:**

Intracellular free calcium, protein phosphorylation, immunostaining of tissues/cells, and multiplex secretion assay.

**Results:**

Similar to immune cells, anaphylatoxin exposure resulted in increases in free cytosolic Ca^2+^, PI3-kinase/Akt activation, FoxP3 and FOXO1 phosphorylation, and cytokine/chemokine secretion. Differential responses were elicited depending on whether C3a and C5a were co-administered or applied consecutively, and response amplitudes in co-administration experiments ranged from additive to driven by C5a (C3a + C5a = C5a) or being smaller than those elicited by C3a alone (C3a + C5a < C3a).

**Conclusion:**

We suggest that this combination of integrative signaling between C3aR and C5aR helps the RPE to precisely adopt its immune regulatory function. These data further contribute to our understanding of AMD pathophysiology.

## Introduction

The retinal pigment epithelium (RPE) is a monolayer of pigmented cells located between the light-sensitive photoreceptors and the fenestrated endothelium of the choriocapillaris (1). The RPE’s central role in retinoid metabolism and outer segment phagocytosis makes it a close interaction partner of the photoreceptors in visual function. It forms a tight epithelium that separates the neural retina from the blood stream. The RPE, which is regarded as part of the outer blood–retina barrier (1, 2), also actively establishes a barrier for immune reactions by forming, on one hand, a physical barrier for immune cells and, on the other hand, by secreting immunomodulatory factors as well as expressing surface receptors to interact with immune cells. Due to the expression of these immunomodulatory mediators, the RPE is regarded as an “educational” or “immunoregulatory” gate, since immune cells that pass through the fenestrated capillaries of the choroid, upon interacting with the outer blood–retina barrier, are skewed toward a regulatory and pro-resolving phenotype (3).

A coordinated immune suppression requires the RPE’s ability to “sense” immune or inflammatory activities. For that purpose, the RPE expresses a large variety of plasma membrane receptors in a very similar manner to that of immune cells, including Toll-like receptors (4) and cytokine receptors such as the receptor for tumor-necrosis factor-α (5) and the CXC-chemokine receptor 4 (6–8). In addition, the native RPE expresses complement receptors C3aR and C5aR to respond to the anaphylatoxins C3a and C5a (9–11). Anaphylatoxins are soluble components produced as part of the activation of the common pathway of the complement system, which culminates in the generation of the cell-killing terminal complement complex (TCC) or membrane attack complex (MAC). A variety of complement-induced functional changes in the RPE has been reported. C5a induces vascular endothelial growth-factor-A (VEGF-A) production (12) and serves as a priming signal for the formation of the NLRP3 inflammasome (13), and sub-lytic concentrations of the TCC/MAC induce VEGF-A secretion by the RPE (14–17). The ability of the RPE to modulate complement activation on its cell surface, preventing a lytic as well as rapidly terminating a sub-lytic MAC attack, is due to the RPE expressing or recruiting various complement inhibitors to its cell surface (18) and is supported by our recent findings demonstrating that normal human serum (NHS) as a source of complement is unable to form a lytic pore in the RPE cell plasma membrane (19). Instead of forming a membrane pore to allow Ca^2+^ to enter the RPE cell, complement activation increases intracellular free Ca^2+^ as a second messenger by activating ion channels, among them the Ca^2+^-dependent K^+^ channel Maxi-K and the voltage-dependent L-type Ca^2+^ channel. The latter becomes constitutively activated by phosphorylation and mediates VEGF-A secretion by the RPE (15). Thus, taken together, the three major biological effectors of the complement cascade, C3a, C5a, and TCC, appear to contribute differentially to complement-evoked RPE cell signaling. Finally, the complement–RPE interaction has attracted considerable attention in research on the pathomechanisms of age-related macular degeneration (AMD). Evidence derived from genetic analyses revealed that polymorphisms in genes for complement factors are associated with an increased risk for developing AMD (20–23). Furthermore, proteomics and immunohistochemical analyses demonstrated accumulation of complement proteins, including C3a and C5a, in drusen that are localized between the RPE and Bruch’s membrane (24–26), indicating that anaphylatoxin/RPE interactions are also involved in AMD pathogenesis.

Intracellular anaphylatoxin signaling mechanisms in the RPE have so far not been investigated in detail. Recent data on anaphylatoxin signaling in T-cells revealed new functional roles for the complement system (27, 28), whereby stimulation of anaphylatoxin receptors in the plasma membrane moves T-cell differentiation toward a Th1 phenotype by phosphorylation of forkhead box protein O1 (FoxO1). This leads to a reduction of forkhead box P3 (FoxP3) expression, a transient marker for activated T-cells and a permanent marker for regulator (Treg) T-cells (29–32). In T-cells, C3a and C5a show additive effects on FoxP3 suppression. Furthermore, in those cells, an intracellular function for complement C3a was discovered (28, 33, 34).

In this study, we hypothesize that anaphylatoxins lead to changes in the functional phenotype of RPE cells rather than resulting in degeneration. Our study reports that the signaling pathways initiated by C3a or C5a lead to changes in Ca^2+^ signaling, triggering kinase-dependent pathways, activation of transcription factors, and alterations in gene expression and cytokine secretion. Importantly, we uncovered interactions between C3a- and C5a-mediated signaling pathways that are not simply additive, leading to the activation of transcription factors, among them FoxP3 and FOXO1, as well as altered interleukin-8 (IL-8) secretion.

## Materials and Methods

### Cell Culture

Human RPE cells (ARPE-19, LGC Standards/ATCC and primary human RPE cells) were maintained in DMEM/Ham’s F12 (Sigma-Aldrich) supplemented with 10% fetal bovine serum (FCS; Biochrom or Thermo Fisher Scientific) and 0.5% penicillin/streptomycin (Biochrom or Thermo Fisher Scientific) at 37°C in a humidified atmosphere. Cells were switched to serum-free medium 24 h prior to experiments.

The isolation of primary RPE cells from human cadaver eyes was approved by the ethics committee of the Clinic of the University of Munich, Germany, and the methods for securing the human tissue were compliant with the Declaration of Helsinki. Donor eyes were obtained from the Eye Bank of the Eye Hospital (LMU München, Germany) and processed within 24 h after death. Isolation of human RPE cells was performed as described (35).

### Induction of Experimental Uveitis in Rats

Lewis rats (Lew/Orl Rj) were bred and maintained under pathogen-free conditions with water and food *ad libitum* and used for experiments at the age of 6–8 weeks. All animal experiments were approved by the Review Board of the Regierung von Oberbayern (Permit-Number 55.4-1-54-2531-225-2015) and conformed to the ARVO Statement on the Use of Animals in Ophthalmic and Vision Research. Animals were immunized subcutaneously in both hind legs with 100 µl emulsion containing 15 µg peptide R14 (human IRBP aa 1169–1191; Polypeptide Laboratories) and CFA, fortified with *Mycobacterium tuberculosis* strain H37RA (BD Biosciences) to a final concentration of 2.5 mg/ml. Uveitis was scored clinically by ophthalmoscopy to determine inflammation as described (36).

### Immunofluorescence Staining of Tissue Sections

For histology, rat eyes were embedded in Tissue Tec OCT (Paesel and Lorey) and snap frozen in methyl butane (Merck). Air-dried cryosections (8 µm) were post-fixed in ice-cold acetone for 10 min, stained with hematoxilin (Merck), and graded to obtain a pathology score as described (36). For immunofluorescence staining, acetone-fixed sections were pre-incubated with PBS containing 3% normal rabbit serum and 3% donkey serum for 15 min at room temperature (RT), washed once with PBS, and then incubated with rabbit anti-rat FoxP3 antibody (Novus Biologicals, Abingdon, UK; diluted 1:500 in PBS), mouse anti-rat TCR-ab-FITC clone R73 (eBioscience, diluted 1:40), or mouse anti-rat TCR-gd-FITC clone V65 (Aviva Systems, diluted 1:6) for 1 h at RT in the dark. Control stainings were performed with secondary antibody only. After PBS washes, Cy3-conjugated donkey anti-rabbit IgG(H + L) (Jackson Laboratories) was added (1:100 in PBS) and incubated for 1 h at RT in the dark. The slides were washed, mounted with Entellan (Merck), imaged with a Zeiss Axioskop 2plus (Carl Zeiss), and photographs taken with a Sony CyberShot DSC-S70 3.3 mp digital camera.

### Immunofluorescence Staining of ARPE-19 Cells

ARPE-19 cells were grown in chamber slides overnight. Cells were washed with PBS/1% BSA and fixed in 4% paraformaldehyde for 15 min. Cells were then incubated in FcR blocking buffer [1:10 dilution of Fc receptor blocking reagent (Mittenyi Biotec GmbH) in PBS/1% BSA] for 10 min on ice, followed by incubation with primary antibody mAb C3aR (1:400; Santa Cruz Biotechnology) or C5aR (1:400; BioLegend) for 1 h at RT. After washing, cells were first incubated with goat anti-mouse Alexa Fluor^®^ 488-conjugated secondary antibody (1:600; Thermo Fisher Scientific), followed by incubation with DAPI (Sigma, Munich, Germany) for 15 min and coverslipping. Pictures were taken on an LSM710 microscope (Carl Zeiss AG) using 63x, 1.4 NA, oil immersion, and ZEN2009 software (Carl Zeiss AG).

### Calcium Imaging

Serum-deprived cells grown on 15 mm glass cover slips (8.5 × 10^3^ cells/cm^2^) were incubated for 40 min with 2 µM fura-2/AM (F1221, Invitrogen). The cover slips were placed in a custom-made recording chamber (filled with bath solution consisting of (in mM): 138 NaCl, 5.8 KCl, 0.41 MgSO_4_, 0.48 MgCl_2_, 0.95 CaCl_2_, 4.17 NaHCO_3_, 1.1 NaH_2_PO_4_, 25 HEPES) and imaged using a Zeiss Axiovert 40 CFL inverted microscope (Carl Zeiss AG) equipped with a 40 × oil immersion objective, a Visichrome High Speed Polychromator System (Visitron Systems), and a high-resolution CCD camera (CoolSNAP EZ, Photometrics) as described previously (19). Experiments were carried out by adding C3a (260 nM) and/or C5a agonists (52 nM; Complement Technology, Inc.), nifedipine (10 µM; Tocris Biosciences), LY294002 (50 µM; Cayman Chemical Company), or API-2 (10 µM; Santa Cruz Biotechnology) to the bath solution. Data acquisition and analysis were carried out using the MetaFluor Fluorescence Ratio Imaging Software (Visitron Systems). Fluorescence intensity of Fura-2 was detected at an emission wavelength of 505 nm, while the excitation wavelengths were set to 340/380 nm. Changes in intracellular free Ca^2+^ are all given as ratios of the fluorescence of the two excitation wavelengths (dF/F) and normalized to baseline (ddF/F).

### Western Blotting and Dot Blotting

ARPE-19 cells were grown on transwell plates and stimulated with C3a and/or C5a agonists. Cells were solubilized in RIPA buffer (Thermo Fisher Scientific) containing a cocktail of protease and phosphatase inhibitors (Sigma-Aldrich). Whole cell lysates were clarified by centrifugation (20,000 × *g* for 30 min at 4°C), and samples were quantified (Pierce BCA protein assay reagent kit; Thermo Fisher Scientific). For western blotting, equivalent protein amounts were added to Laemmli sample buffer and boiled. Samples were separated by electrophoresis on a 4–20% Criterion™ TGX™ Precast Gels (Bio-Rad Laboratories, Inc.), and proteins were transferred to a PVDF membrane. For Dot Blotting, lysates containing equivalent amount of total protein were loaded on a 96-well plate (Bio-Dot ^®^ Microfiltration Apparatus; Bio-Rad Laboratories Inc.) and vacuum-transferred onto nitrocellulose membranes. Membranes were incubated with primary antibodies (1:1,000) against Phospho-Akt (Ser 473), Phospho-FOXO1 (Ser 256), Phospho-CREB (Ser 133), β-actin, Phospho-Ca_V_1.3 (all from Cell signaling Technology), or Phospho-FoxP3 (Ser 418; Abgent, Inc.) overnight using β-actin or GAPDH (both from Cell Signaling Technologies) as controls. Proteins were visualized with horseradish peroxidase–conjugated secondary antibodies (Santa Cruz Biotechnology), followed by incubation with Clarity™ Western ECL Blotting Substrate (Bio-Rad Laboratories, Inc.) and chemiluminescent detection. Protein bands or dots were scanned, and densities were quantified using ImageJ software.

### Immunoprecipitation

For immunoprecipitation of calcium channels, ARPE-19 cells were extracted by solubilizing cells in RIPA buffer and whole cell lysates were clarified as described above. Lysates were precleared with 25 µl of Protein A-agarose beads (Cell Signaling Technology) to remove the non-specific binding proteins, and then samples with equivalent protein content were incubated with 1 µg of the anti-Ca_V_1.3 antibody (Alomone Labs) overnight at 4°C with agitation. Next, 150 µl of Protein A-Agarose beads was added to the samples and incubation was continued for 4 h at 4°C. Immunoprecipitated complexes bound to the beads were collected by centrifugation (5,000 × *g* for 10 min at 4°C), washed by resuspension, followed by centrifugation. Finally, each pellet was resuspended in 50 µl of Laemmli sample buffer, boiled, and centrifuged prior to loading. Samples were then separated by electrophoresis, and proteins were transferred to a PVDF membrane and blotted for Ca_V_1.3 as described above.

### Gene Expression Analysis

Retinal pigment epithelium cells were grown to confluency on transwell plates and maintained under serum-free conditions for 24 h before harvesting. A subset of ARPE-19 cells was stimulated with C3a and/or C5a agonists for 24 h. RNA isolation and cDNA synthesis were performed using the RNeasy Mini and Quantitect Reverse Transcription Kit (Qiagen). The mRNA levels of C3, C3aR, C5, FOXO1, and GAPDH (Eurofins Genomics) were measured in triplicates by RT-PCR (Rotor-Gene SYBR Green PCR Kit; Qiagen, Hilden, Germany) on a Rotor-Gene Q (Qiagen); those of C5aR were measured by TaqMan^®^ Gene Expression Assay (Thermo Fisher Scientific). For primers, see Table 1. Quantification of the target genes was carried out using the comparative CT (threshold cycle, ΔΔC_T_) method using Rotor-Gene Q software 2.2.3 (Qiagen) (37).

**Table 1:** Primer characteristics.

Gene	Forward sequence	Reverse sequence
C3	TTCCGATTGAGGATGGCTCG	ATGTCACTGCCTGAGTGCAA
C3aR	GGCTGTCTTTCTTGTCTGCTG	GACTGCCTTGCTTTCTTCCTAA
C5	ACACTGGTACGGCACGTATG	GGCATTGATTGTGTCCTGGG
C5aR1	Hs00704891_s1	
FOXO1	TGCATTTCGCTACCCGAGTT	GTGGCTGACAAGACTTAACTCAA
GAPDH	TCAACGACCACTTTGTCAAGCTCA	GCTGGTGGTCCAGGGGTCTTACT

### Cytokine/Chemokine Secretion

ARPE-19 cells were grown to confluency. After 3 days, cells were switched to serum-free medium for 24 h. Then medium was exchanged with serum-free DMEM/Ham’s F12, to which C3a or C5a or both were added to hexaplicate cultures each for 3 days. Some cultures with or without complement components were additionally supplied with PI3-kinase inhibitor LY294002. Supernatants from hexaplicate cultures (two wells each) were pooled and tested in triplicates by human Bio-Plex bead analysis (Bio-Rad Laboratories, Inc.) and measured using the Bio-Plex 200 (Bio-Rad Laboratories, Inc.). Tested analytes included IL-1beta, IL-1ra, interleukin- 6 (IL-6), IL-8/CXCL8, interleukin-10 (IL-10), IL-12(p70), IFN-gamma, monocyte chemotactic protein-1 (MCP-1)/CCL2, and VEGF.

Monocytes were isolated from blood of three different healthy male donors using indirect magnetic labeling separation (MACS pan monocyte isolation kit, Miltenyi Biotec). 1 × 10^5^ cells were incubated with C3a (3.3 µM), C5a (480 nM), or both in a 96-well plate at 37°C for 20 h. Supernatants were collected, and interleukins (IL-1β, IL-6, and IL-10) were measured using ELISA (Ready-Set-Go^®^ ELISA kits, eBioscience, Inc.).

### FACS Analysis

6 × 10^5^ human primary monocytes were incubated with BSA, C3a (3 µM, Complement Technology, Inc.), or C5a (0.6 µM, Complement Technology, Inc.) for 5 min at 37°C. Cells were then washed with PBS and bound with monoclonal antibody anti-CD88 (anti-C5aR, 1:200, BioLegend) for 1 h on ice. Cells were washed afterward and incubated with corresponding goat anti-mouse Alexa Fluor^®^ 647-conjugated secondary antibody (1:400, Invitrogen) for 30 min on ice in the dark. Cells were washed with PBS and diluted in 300 µl PBS. FACS measurement was carried out with BD LSR II flow cytrometry (BD Biosciences).

### Statistical Analysis

All data are presented as mean values ± SEM or ±SD. Statistical significance was calculated using Mann–Whitney *U* test for Ca^2+^-Imaging analyses and protein secretion analyses. For western blot and gene expression analyses, Student’s *t*-test was used (*p* values *p < 0.05, **p < 0.01, and ***p < 0.005). All calculations were performed in SPSS 22 and Excel 2010.

## Results

### Properties of Anaphylatoxin-Evoked Ca^2+^ Responses by RPE Cells

**Figure 1:**
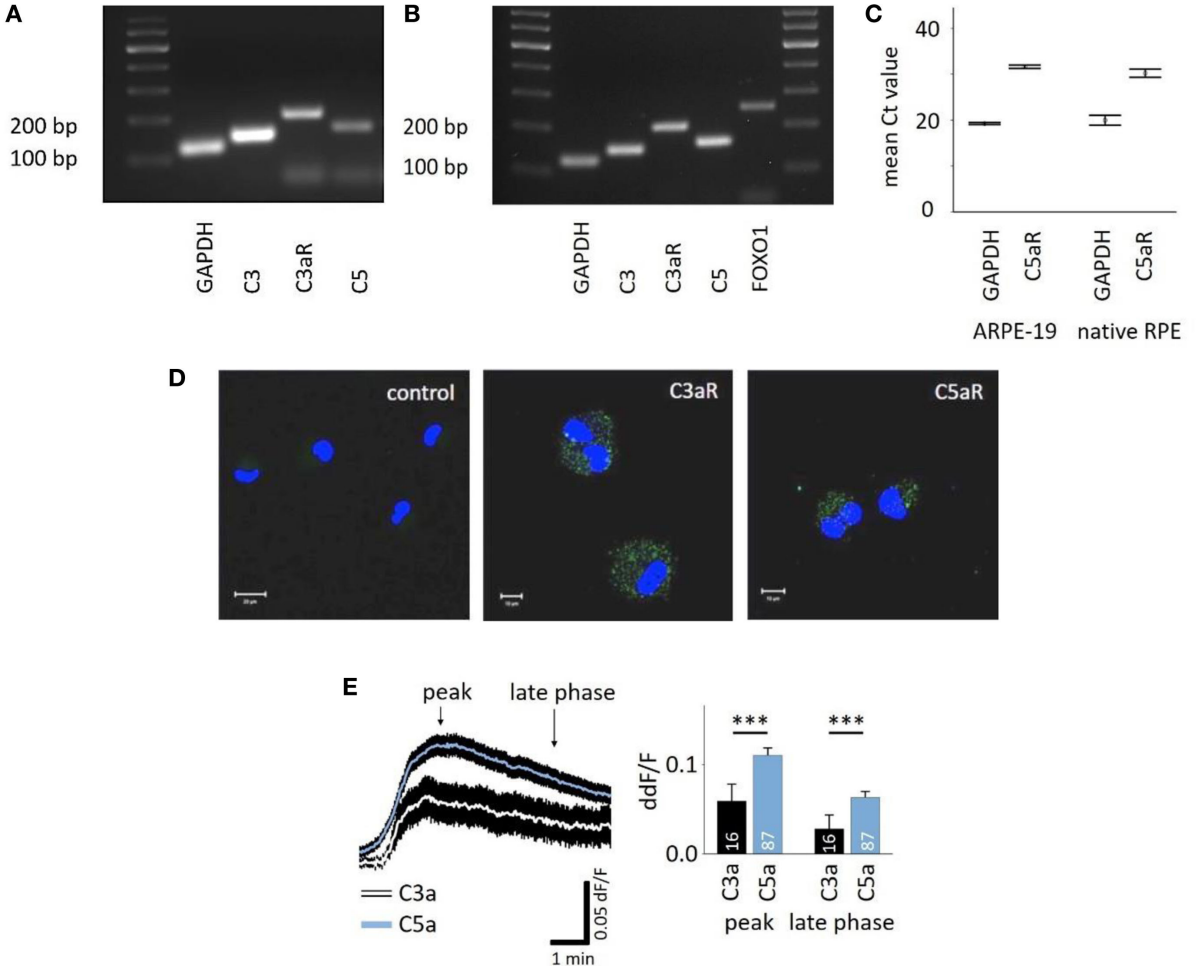
Retinal pigment epithelium (RPE) cells express functional anaphylatoxin receptors C3aR and C5aR. **(A)** RT-PCR from ARPE-19 cells showing the expression of C3, C5, and C3aR mRNA. **(B)** RT-PCR from human native RPE cells showing the expression of C3, C3aR, C5, and FOXO1. **(C)** Mean Ct-values of qPCR from ARPE-19 cells and human native RPE cells showing the expression of C5aR1. **(D)** Immunofluorescence staining of ARPE-19 cells for surface expression of C3aR and C5aR with primary monoclonal antibodies detecting C3aR or C5aR1 and secondary green fluorescent antibody (Alexa Fluor^®^ 488). Control: secondary antibody only. Nuclear DNA is stained with DAPI. Size marker = 10 µm. **(E)** Left panel: time course of anaphylatoxin-evoked Ca^2+^ transients in ARPE-19 cells. The black rim indicates the SEM. C3a/C5a are added at the start of the displayed Ca^2+^ transient. Arrows mark time points taken for statistical analysis: the peak and the sustained phase (3 min after peak = late phase). Right panel: statistical analysis of the effects of C3a and C5a application on intracellular free Ca^2+^ in ARPE-19 cells at peak and late phases, normalized to baseline ratio (ddF/F). Anaphylatoxins were applied at concentrations of 260 nM (C3a) and 52 nM (C5a). Please note that all other figures depicting Ca^2+^ transients are laid out and analyzed in the fashion described here. Data are mean + SEM from two to six independent experiments; number of cells is indicated in the bars, ***p < 0.005 (Mann–Whitney *U* test).

C3aR and C5aR mRNAs are expressed in the RPE cell line ARPE-19 (Figures 1A,C) as well as primary human RPE cells (Figures 1B,C), and protein expression was confirmed in ARPE-19 cells (Figure 1D). The most common intracellular response triggered by the engagement of C3aR or C5aR is the mobilization of calcium. Here, we demonstrated functional signaling of both receptors in ARPE-19 cells in response to the application of C3a and C5a, which resulted in a biphasic increase in intracellular free Ca^2+^, composed of an initial peak after ~90 s and a plateau phase that is reached after ~3 min (Figure 1E, left panel). The response to C5a reached a 2x larger peak and plateau phase than the C3a response (Figure 1E, right panel). *In vivo*, the RPE is separated from the blood stream by Bruch’s membrane, making it impossible to predict the concentration of anaphylatoxins that potentially reach the RPE. Thus, we adjusted the anaphylatoxin contractions used for experiments to the known receptor binding constants. The anaphylatoxin concentrations used (260 nM C3a and 52 nM C5a) were close to saturation (38, 39).

**Figure 2:**
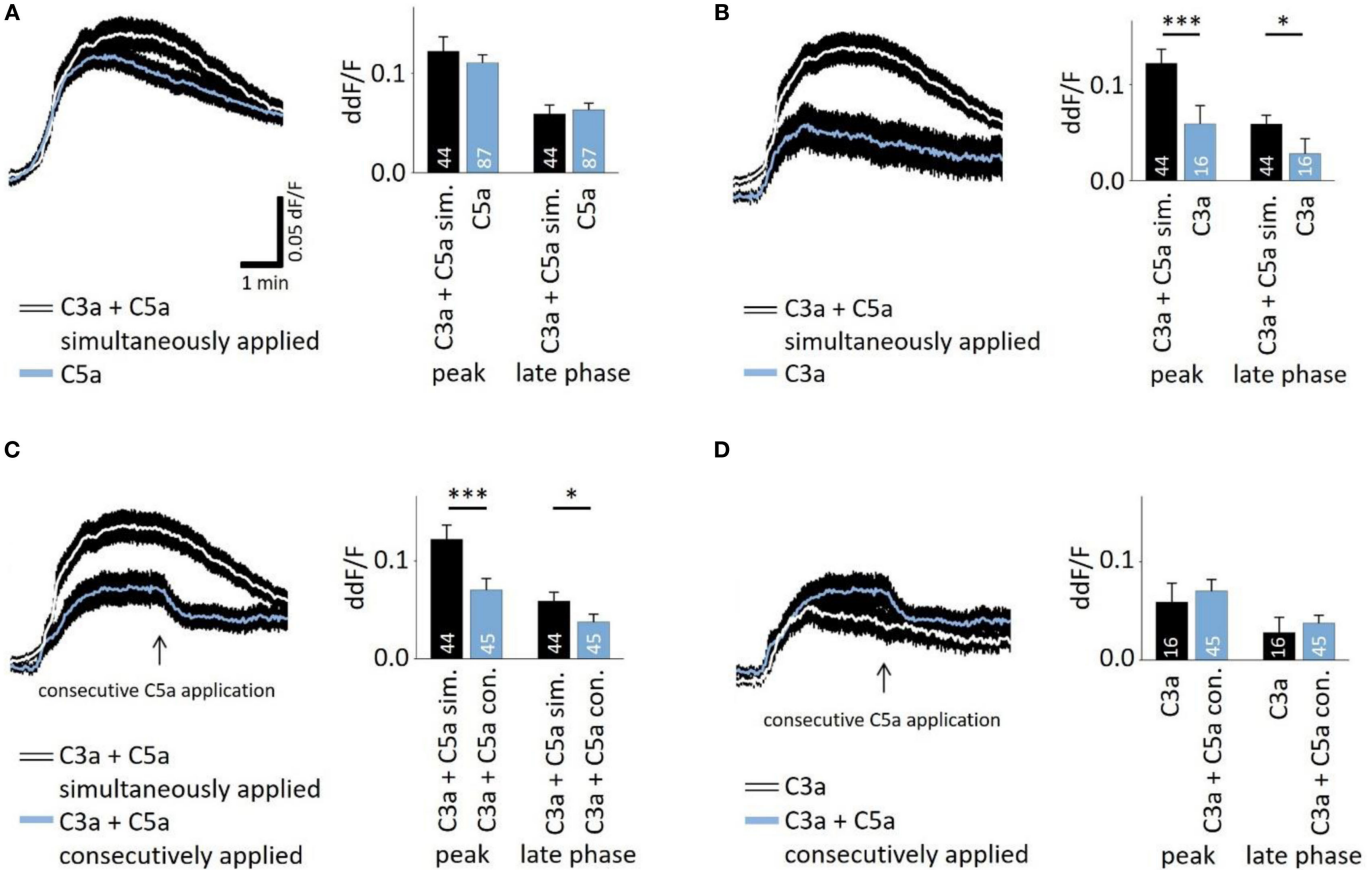
Interactive signaling between C3a and C5a stimulation observed in calcium transients. The time courses of anaphylatoxin-evoked Ca^2+^ transients in ARPE-19 cells were examined in cells exposed to anaphylatoxins alone or in combination (left panels), and peak and late phase amplitudes were analyzed (right panels). Simultaneous **(A,B,C)** versus consecutive **(C,D)** applications were tested. **(A)** Single application of C5a compared to the simultaneous co-application of C3a and C5a. **(B)** Single application of C3a compared to the simultaneous co-application of C3a and C5a. **(C)** Simultaneous co-application of C3a and C5a compared to consecutive application, where C5a was applied after C3a when the Ca^2+^ transient has reached a steady state indicated by arrow. **(D)** Single application of C3a compared to consecutive co-application of C3a followed by C5a. Anaphylatoxins were applied at concentrations of 260 nM (C3a) and 52 nM (C5a). Data are presented as mean values + SEM. Numbers of cells tested in two to seven independent experiments is indicated in the bars, *p < 0.05, ***p < 0.005 (Mann–Whitney *U* test).

In the presence of sufficient C3 and C5, C5a production follows that of C3a in very short succession during the activation of the cascade. To investigate whether there is an interaction of C3a- and C5a-dependent signaling, C3a and C5a were added simultaneously to cells. Co-application also led to a biphasic elevation of intracellular free Ca^2+^ (Figures 2A–C), following the same temporal profile like individual applications. However, the response to simultaneously applied anaphylatoxins was not significantly different compared to that of C5a alone (Figure 2A, left panel), but it was significantly higher when compared to the single C3a application (Figure 2B). These data suggest that the two anaphylatoxin receptors do not act in an additive manner, but the response is primarily driven by C5aR activity alone. Since the two anaphylatoxins are produced in succession rather than simultaneously, we applied them in sequence: C3a was applied first, and after the Ca^2+^ response reached a steady state, C5a was applied (Figure 2C). Interestingly, under these conditions, C5a significantly reduced the steady-state Ca^2+^ level that has been reached by the previous C3a application (ddF/F at time point of C5a application: 0.763; ddF/F 30 s after C5a application: 0.746; *p* < 0.001). The consecutive signaling of C3a and C5a reached a plateau phase that was significantly lower than that observed with simultaneous application (Figure 2C). The response to the consecutive application of C3a and C5a was not significantly different from that of the single C3a application (Figure 2D). Incubation of human monocytes with C3a did not change C5aR surface expression measured by means of FACS analysis (Figure S1 in Supplementary Material). This second set of data further indicates that C3aR and C5aR do not act in an additive manner or in concert but rather regulate each other, with C3aR engagement presumably preventing a future C5aR response.

### Intracellular Signaling Mechanisms of Anaphylatoxin-Evoked Ca^2+^ Responses

**Figure 3:**
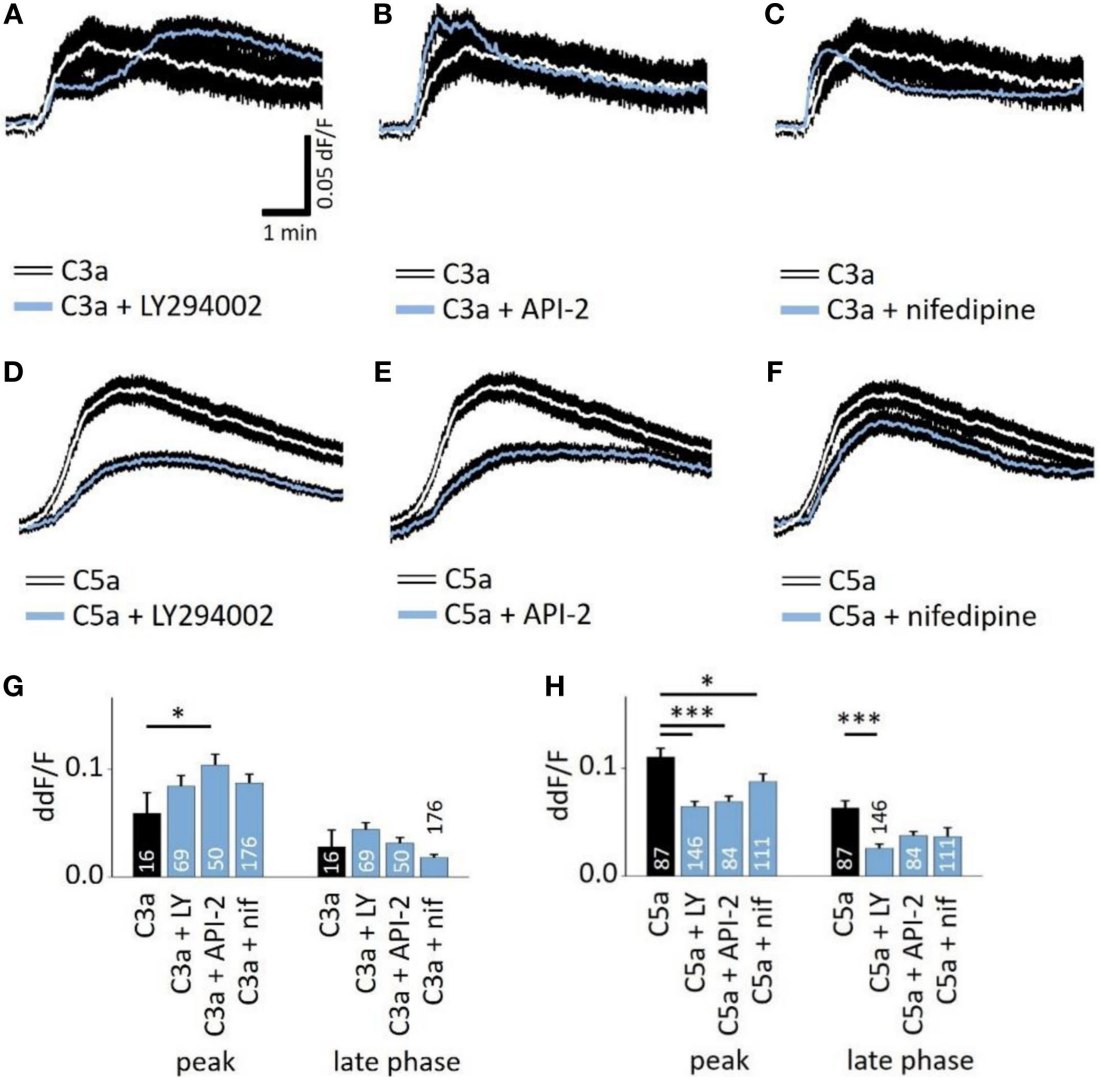
Role of PI3-kinase and Akt in anaphylatoxin-evoked Ca^2+^ transients. **(A,D)** Effect of the PI3-kinase blocker LY294002 (50 µM) on C3a-evoked **(A)** or C5a-evoked **(D)** Ca^2+^ transients in ARPE-19 cells. **(B,E)** Effect of the Akt blocker API-2 (10 µM) on C3a-evoked **(B)** or C5a-evoked **(C)** Ca^2+^ transients in ARPE-19 cells. **(C,F)** Effect of the L-type channel blocker nifedipine (10 µM) on C3a-evoked **(C)** or C5a-evoked **(F)** Ca^2+^ transients in ARPE-19 cells. **(G,H)** Statistical comparison of blocker application effects on C3a-evoked **(G)** and C5a-evoked **(H)** Ca^2+^-transients at the peak and late phases. Anaphylatoxins were applied at concentrations of 260 nM (C3a) and 52 nM (C5a). **(A–F)** Black rim indicates SEM. **(G,H)** data are mean + SEM, number of cells as indicated in the bars from six to nine independent experiments, *p < 0.05, ***p < 0.005 (Mann–Whitney *U* test).

Next, we studied whether the anaphylatoxin-evoked Ca^2+^ responses involve known mechanisms of the anaphylatoxin receptor downstream signaling cascades. In immune cells, C3aR signals through the pertussis toxin-sensitive G-proteins, and C5aR signals through the pertussis toxin-sensitive alpha units Gαi2 or the pertussis toxin-insensitive Gα16 (28). Subsequent downstream signaling includes mainly the activation of PI3-kinase, followed by the activation of Akt (27), leading to calcium mobilization from intracellular stores (28). In RPE cells, we have shown that NHS leads to strong activation of L-type Ca^2+^ channels, allowing for the influx of extracellular calcium (19). In order to study these potential mechanisms of anaphylatoxin signaling, we used different pathway-specific blockers. The PI3-kinase blocker LY294002, the Akt blocker API-2, or the L-type Ca^2+^ channel blocker nifedipine was added to the cells prior to stimulation. LY294002 is a pan-isoform PI3K blocker, which was used since the specific PI3K isoforms involved in ion channel regulation have not yet been identified. LY294002, API-2, and nifedipine had little effect on the C3a-evoked Ca^2+^ responses during both the peak and late phases (Figures 3A,C,G), although the Akt blocker API-2 surprisingly increased the C3a-evoked Ca^2+^ response at the peak phase (Figures 3B,G). In contrast, LY294002 decreased the Ca^2+^ response at both the peak and plateau phases upon application of C5a (Figures 3D,H). In addition, the Akt blocker API-2 reduced the C5a-evoked Ca^2+^ rises, although only during the peak but not the plateau phase (Figures 3E,H, peak: *p* = 0.001). Thus, PI3-kinase and Akt1 signaling participate in C5a-evoked Ca^2+^ responses, in which both contribute to the peak, but only PI3-kinase contributes to the plateau phase. Finally, we have previously shown that L-type Ca^2+^ channels partly contribute to the plateau phase of the Ca^2+^ signal in response to NHS (19). Furthermore, PI3-kinase is known to stimulate L-type channels (40). However, in the presence of nifedipine, the C3a-evoked response was unaffected, whereas the C5a-evoked Ca^2+^ response was weakly reduced with a small but significant reduction at the peak, but not at the plateau phase (Figures 3F,H).

**Figure 4:**
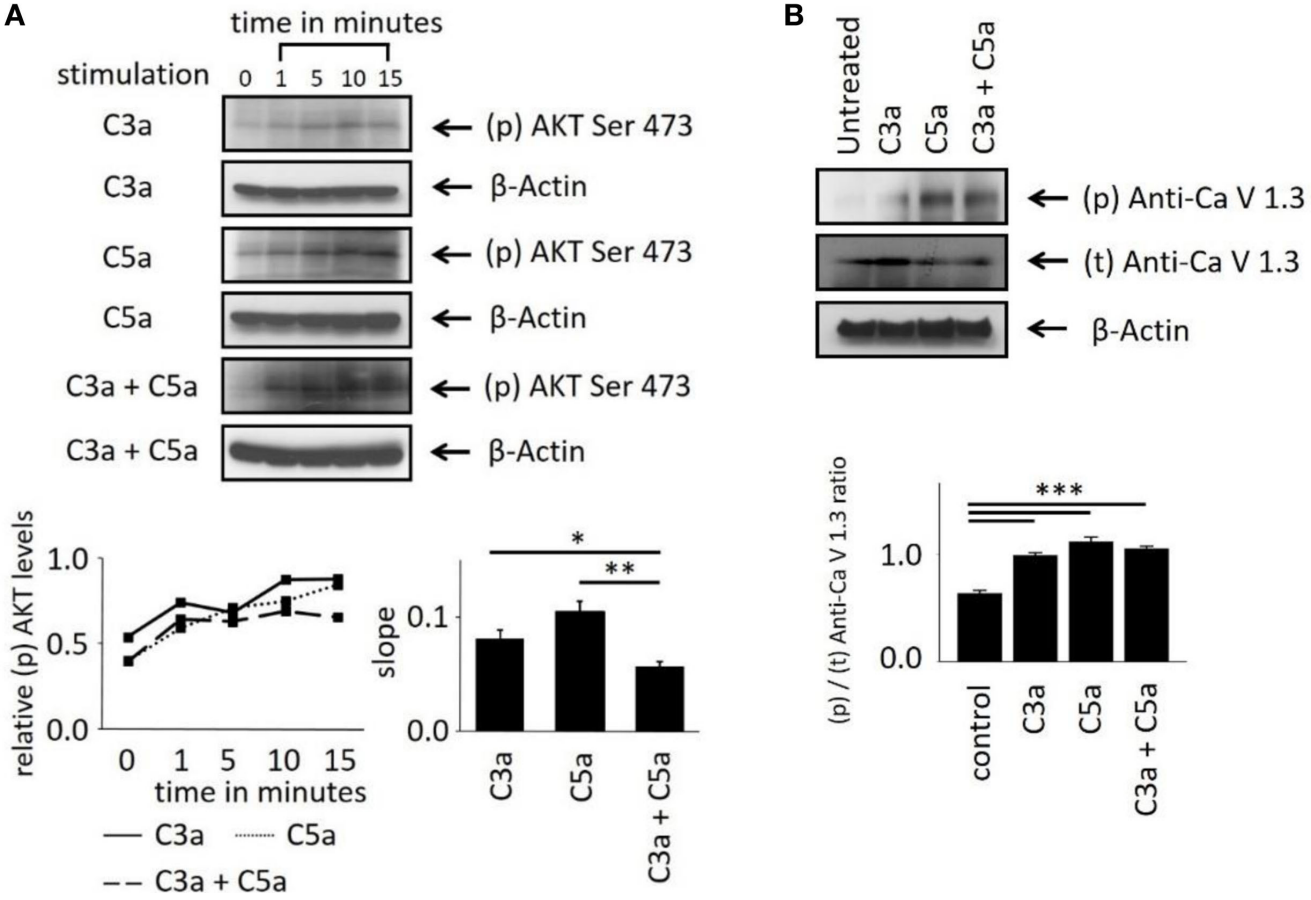
Effects of anaphylatoxins on Akt and L-type Ca^2+^ channel phosphorylation in ARPE-19 cells. **(A)** Akt phosphorylation at serine in position 473 was examined in ARPE-19 cells treated with C3a, C5a, or the combination of C3a/C5a at 1, 5, 10, and 15 min and compared to unstimulated (designated as 0 min) cells. Akt phosphorylation levels were normalized to β-actin (relative p-Akt levels), and slopes of the time course were determined. **(B)** L-type Ca^2+^ channel (Ca_V_1.3 subunit) phosphorylation was examined at the 15-min time point after immunoprecipitation with anti-Ca_V_1.3 and blotting for phosphor-tyrosine. Ca_V_1.3 phosphorylation levels were normalized to β-actin (relative Ca_V_1.3 levels). Anaphylatoxins were applied at concentrations of 300 nM (C3a) and 50 nM (C5a). Data are mean + SEM, *n* = 3, *p < 0.05, **p < 0.01 (Student’s *t*-test).

These Ca^2+^ imaging data indicate the involvement of the central intracellular signaling molecule Akt in anaphylatoxin-evoked Ca^2+^ responses. To confirm the conclusions derived from our pharmacological interventions, we investigated whether Akt is activated and phosphorylated in response to the anaphylatoxins. Phosphorylation of Akt at serine 473, required for full activation of its kinase activity, was determined by western blot analysis. The ratios of phosphorylated Akt to β-actin were calculated, and slopes of change were determined (Figure 4A). C3a, C5a, and combined C3a/C5a application induced Akt phosphorylation significantly after 15 min (Figure 4A); however, when C3a and C5a were applied simultaneously, Akt phosphorylation was reduced below the levels obtained by single application of either C3a or C5a. Thus, although each anaphylatoxin activated Akt, in combination, the activation of Akt was reduced. These results further confirm the non-additive C3aR and C5aR signaling pathways.

Studying the phosphorylation of the L-type Ca^2+^ channel subunit Ca_V_1.3 under anaphylatoxin stimulation revealed (Figure 4B) an increase in Ca_V_1.3 phosphorylation upon C3a, C5a, or combined C3a/C5a application after 15 min (Figure 4B). Ca_V_1.3 phosphorylation was increased upon C5a compared to C3a, but combined C3a and C5a application resulted in phosphorylation levels lower than those with C3a alone. These data are in agreement with our previous experiments using complement-sufficient NHS, which revealed Ca_V_1.3 phosphorylation upon serum exposure and again suggest the presence of competing signaling pathways between C3aR and C5aR activation.

### Anaphylatoxin-Dependent Changes in the Transcription Factor Activation in ARPE-19 Cells

**Figure 5:**
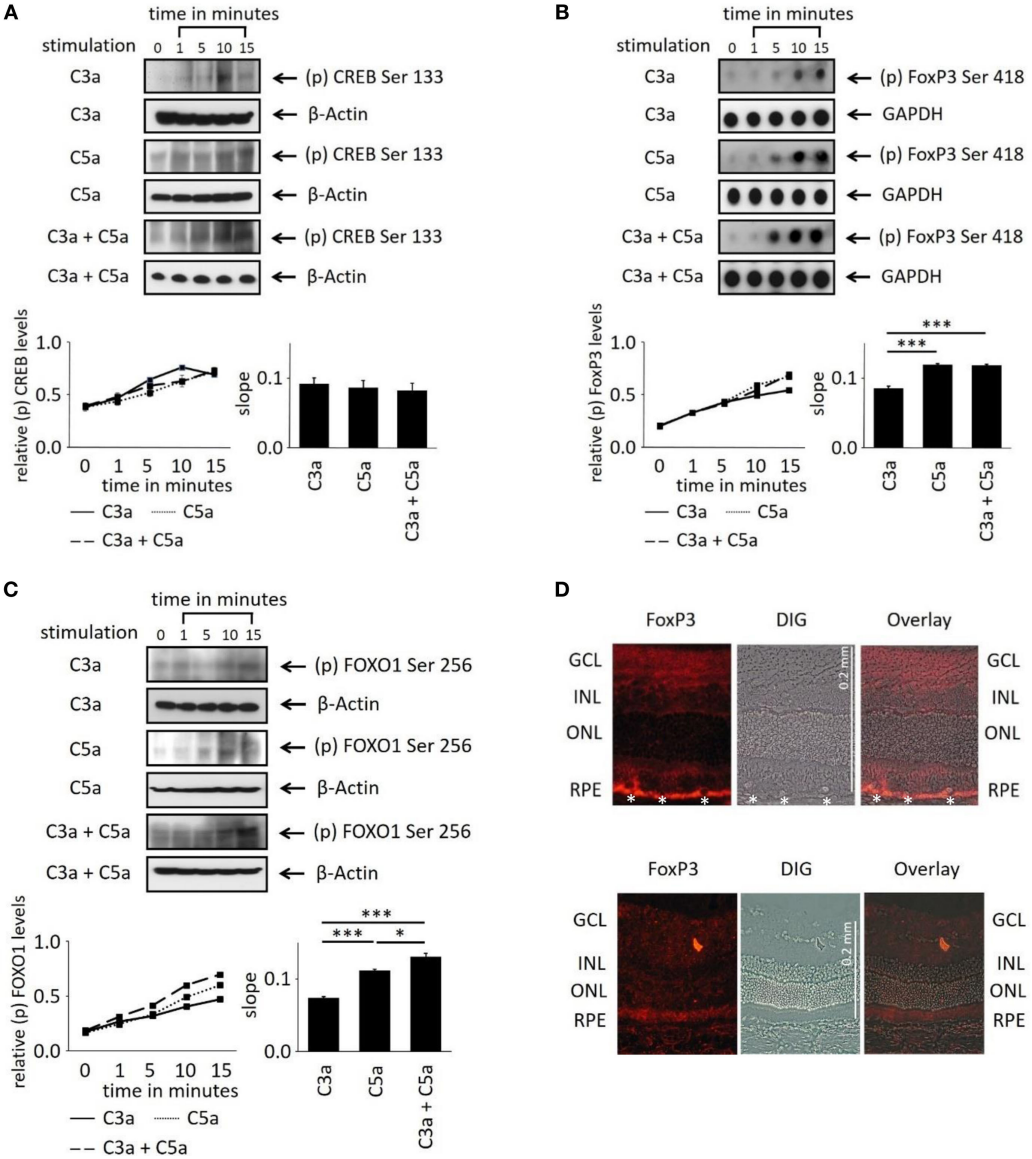
Effects of anaphylatoxins on transcription factor phosphorylation in ARPE-19 cells. **(A–C)** Protein phosphorylation of **(A)** CREB (Ser133); **(B)** FoxP3 (Ser418); and **(C)** FOXO1 (Ser256) was examined in ARPE-19 cells treated with C3a, C5a, or the combination of C3a/C5a at 1, 5, 10, and 15 min. Phosphorylation levels were normalized to β-actin (relative p-protein levels), and slopes of the time course were determined. Anaphylatoxins were applied at concentrations of 300 nM (C3a) and 50 nM (C5a). Data are mean + SEM, *n* = 3, *p < 0.05, ***p < 0.001 (Student’s *t*-test). **(D)** Immunofluorescence staining of FoxP3. Upper panel: Lewis rat (albino) eye with experimental uveitis; lower panel: normal rat eye. Asterisks mark immune cells infiltrating the retina from the choroid through the retinal pigment epithelium (RPE) in the eye with uveitis (clinical score of 2, histological score of 1).

In order to evaluate the longer term impact of anaphylatoxins on RPE function, we investigated changes in transcription factor activation. Since C3a and C5a trigger Ca^2+^ elevations, an increased phosphorylation of the transcription factor Ca^2+^-dependent CREB (cAMP/Ca^2+^ response element binding protein) (41, 42) was expected. C3a or C5a or the combination of C3a/C5a significantly increased CREB phosphorylation over a time course of 15 min (Figure 5A); however, the degree of phosphorylation or the slope did not differ between C3a, C5a, or combined C3a/C5a stimulation.

Activation of T-cells *via* the anaphylatoxin receptors results in the expression and activation of transcription factors FoxP3 and/or FOXO1 (43). Surprisingly, FoxP3 and FOXO1 expression was identified in ARPE-19 cells by dot-blot or western blot analysis (Figures 5B,C). FoxP3 is regarded as a marker of regulatory T-cells and is transiently expressed in activated human and rat effector T-cells (30, 44). FoxP3 protein expression in RPE cells was confirmed by immunohistochemistry in rat retina. FoxP3 was identified in the RPE of rat eyes with uveitis, but not in RPE cells of healthy control retinas (Figure 5D). Immunohistochemistry for alpha/beta and gamma/delta T-cell receptors confirmed that the detected FoxP3 protein was indeed localized to RPE and not to infiltrating T-cells (Figure S2 in Supplementary Material). During harvesting of the eyes, the choroidal blood vessels in the eyes are drained due to the persisting intraocular pressure; hence, no TCR-positive cells can be observed in the choroid unless there is focal choroidal inflammation. Eyes were collected after resolution of the peak of inflammation, when infiltrating T-cells are a rare event. Nevertheless, some remaining T-cells were still detected in the retina (Figure S2B in Supplementary Material), which did not express FoxP3, suggesting that they were neither activated nor regulatory T-cells.

Next, we asked whether anaphylatoxin receptor stimulation leads to activation of FoxP3 and/or FOXO1 in ARPE19 cells. Phosphorylation at serine 418 affects the transcriptional activity of FoxP3, whereas phosphorylation of serine 256 is critical for FOXO1. C3a, C5a, and combined C3a/C5a application significantly induced FoxP3 phosphorylation after 15 min (Figure 5B).

FoxP3 phosphorylation was rapidly induced by C3a and even more so by C5a (Figure 5B). The combination of C3a and C5a did not further increase FoxP3 phosphorylation than triggered by C5 alone (Figure 5B). FOXO1 phosphorylation was also induced by either C3a, C5a, or combined C3a/C5a application (Figure 5C). Stimulation with C5a induced higher FOXO1 phosphorylation, resulting in steeper slopes than that induced by C3a (*p* < 0.001). The combined C3a/C5a application increased FOXO1 phosphorylation even further. In conclusion, the anaphylatoxins induced activation of transcription factors FoxP3 and FOXO1 *via* their respective receptors. Since CREB was also activated (Figure 5A), the anaphylatoxin-dependent changes in gene expression are likely Ca^2+^-dependent.

### Anaphylatoxin-Dependent Changes in ARPE-19 Cell Function

**Figure 6:**
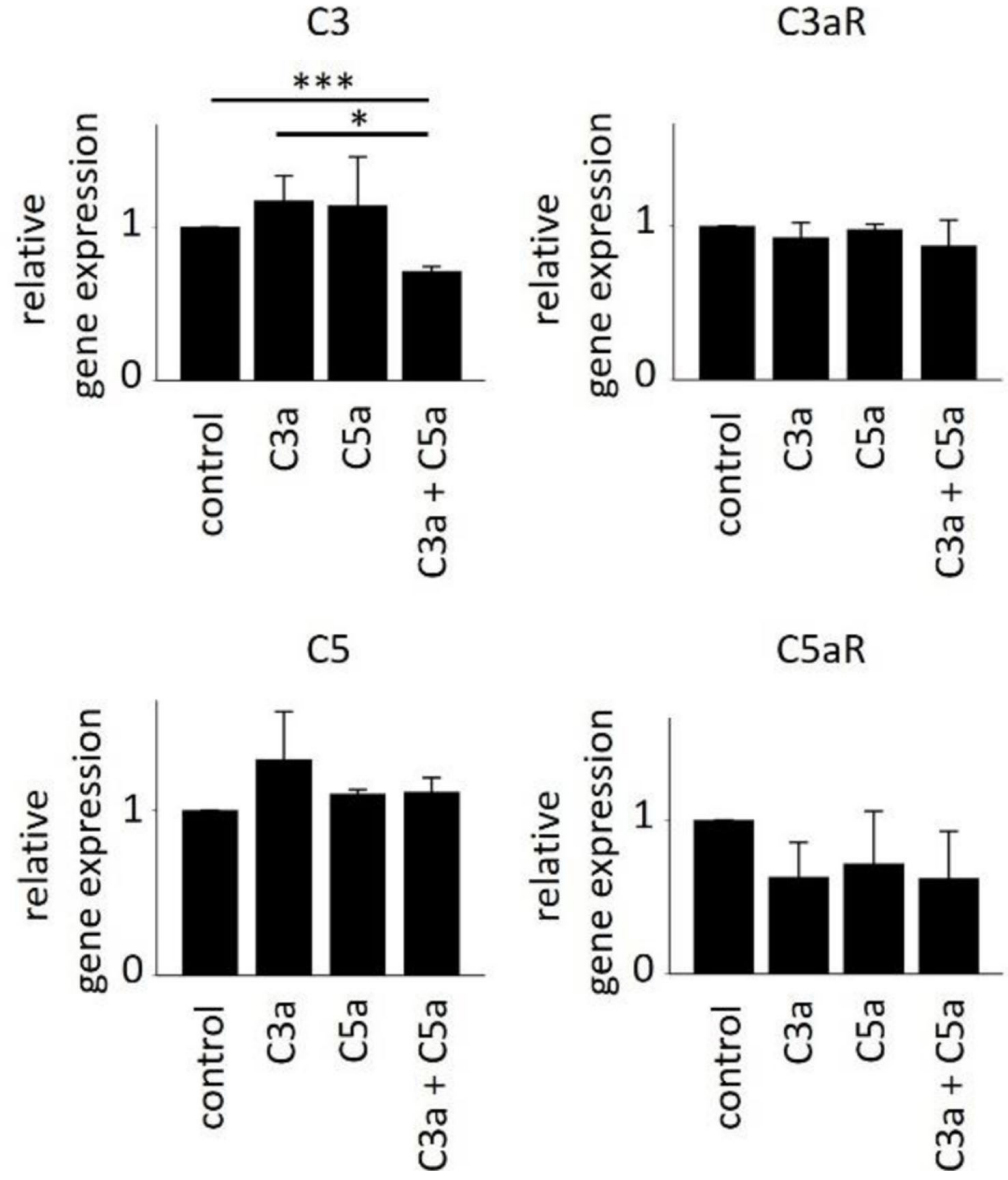
Effects of anaphylatoxins on gene expression of C3, C3aR, C5, and C5aR in ARPE-19 cells. Quantitative PCR analysis of the anaphylatoxin-dependent regulation of complement factor gene expression for C3 (upper left panel), C3aR (upper right panel), C5 (lower left panel), and C5aR (lower right panel). Anaphylatoxins were applied at concentrations of 52 nM (C3a) and 52 nM (C5a). Data are expressed as mean values + SEM, *n* = 3, *p < 0.05, ***p < 0.005 (Student’s *t*-test).

In order to further investigate the consequences of anaphylatoxin receptor signaling, we studied changes in mRNA expression of selected target genes as well as chemokine and cytokine secretion. As we have shown previously that C3aR activation controls gene expression of C3 under pathological conditions (45), we examined anaphylatoxin-dependent changes in gene expression of C3 and C5 as well as the expression of C3aR and C5aR by qPCR (Figure 6). Overall, anaphylatoxin stimulation of ARPE cells did not affect the expression of these four genes, apart from the combined application of C3a and C5a, which decreased C3 expression when compared with the unstimulated control or stimulation by C3a alone.

**Figure 7:**
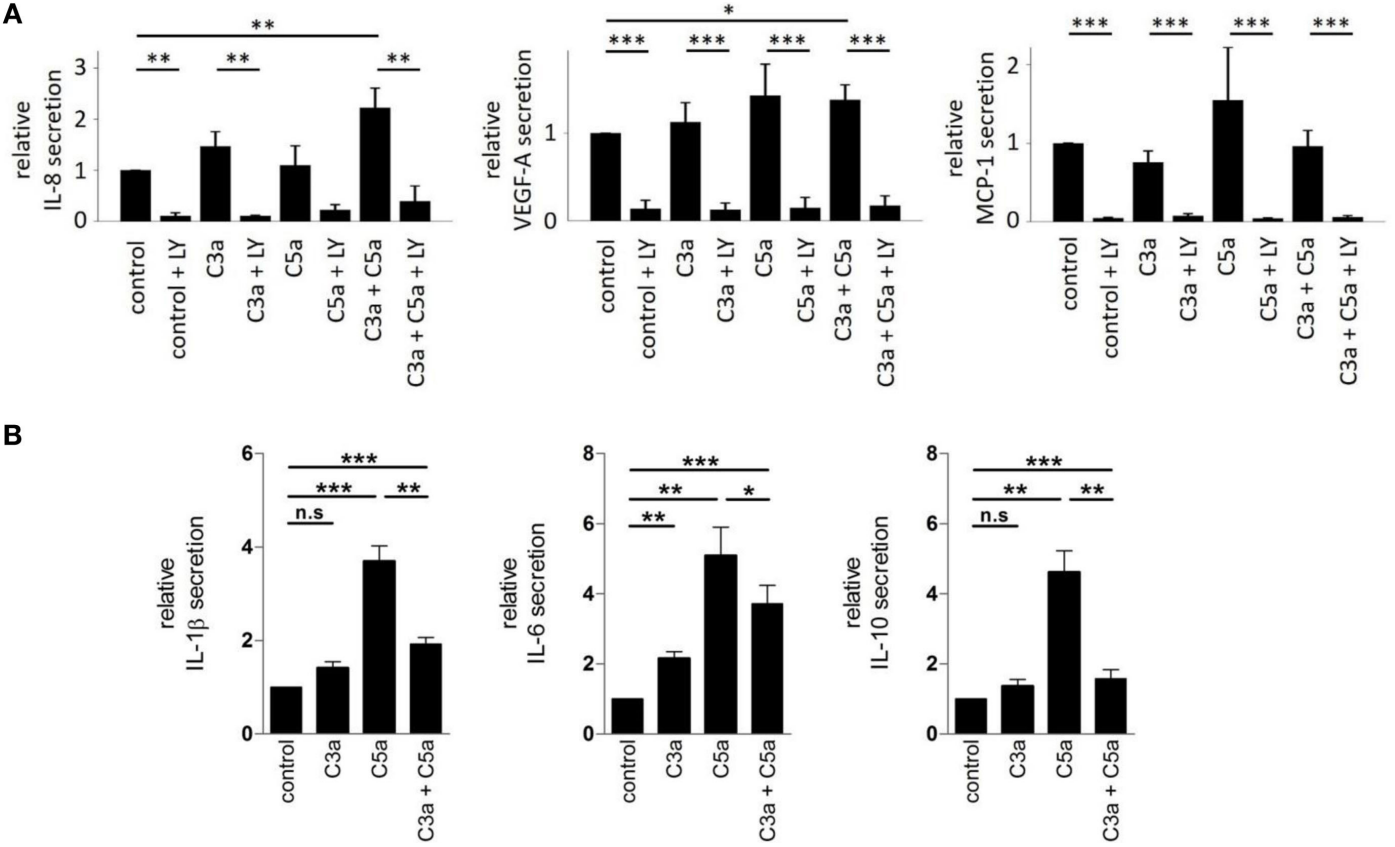
Effects of anaphylatoxins on cytokine and growth factor secretion by ARPE-19 cells and human monocyctes. **(A)** Secretion of interleukin-8 (IL-8), vascular endothelial growth-factor-A (VEGF-A), and monocyte chemotactic protein-1 (MCP-1) by ARPE-19 cells stimulated with C3a, C5a, or both was examined, relative to the values measured in the absence of anaphylatoxins (control; control was arbitrarily set as 1). Co-application of LY294002 (50 µM) with the different anaphylatoxins (C3a: 260 nM; C5a: 52 nM, or combined C3a/C5a: 260/52 nM) was used to test whether constitutive or induced secretion required PI3-kinase activation. **(B)** Secretion of interleukin-1 beta (IL-1β), interleukin-6 (IL-6), and interleukin-10 (IL-10) was tested in human monocytes stimulated with C3a (3.3 µM), C5a (480 nM), or both when compared with buffer alone. Level of cytokine induction by C5a was arbitrarily set as 1. Data are expressed as mean values ± SD; *n* = 3; *p < 0.05; **p < 0.01, ***p < 0.005 [**(A)** Mann–Whitney *U* test, **(B)** Student’s *t*-test].

We further examined the secretory phenotype of ARPE-19 cells after treatment with anaphylatoxins (Figure 7A) by investigating the secretion of cytokines using multiplex technology. Of the analytes investigated, only secretion of IL-8, MCP-1, and VEGF could be verified. A significant increase in IL-8 and VEGF-A secretion was induced by co-administration of C3a and C5a, but not when each anaphylatoxin was used alone (Figure 7A); secretion of MCP-1 was not affected by anaphylatoxin treatment. The secretion of MCP-1, VEGF-A, and IL-8, whether constitutive or anaphylatoxin-induced, was almost completely blocked by the application of LY294002, indicating a dependence of their secretion on the activity of PI3-kinase. Taken together, C3a- and C5a-induced changes in intracellular Ca^2+^, FOXO1, and FoxP3 activation and secretion of cytokines suggest an immune cell-like profile of ARPE-19 cells.

Next, we asked whether similar C3aR- and C5aR-mediated signaling interactions can also be identified in immune cells such as monocytes. In these experiments, we used higher anaphylatoxin concentrations than those in experiments with RPE cells. Reasons for that are monocytes are directly exposed to blood-derived anaphylatoxins, which reach higher concentrations. Thus, we calculated the anaphylatoxin concentrations based on the published C3 and C5 concentrations in human serum. We determined the secretion of IL-1β, IL-6, and IL-10 from freshly isolated human blood monocytes, which are known to be regulated by anaphylatoxins (46) (Figure 7B). Secretion of the three cytokines was increased upon application of C3a or C5a alone, with a 2–3x stronger increase by C5a. Co-application of C5a together with C3a suppressed the secretion of IL-1β or IL-10 to a level below that induced by stimulation with C5a only, but not below the levels obtained with C3a stimulation alone. Altogether, ARPE-19 and monocytes showed similar activation pattern by the anaphylatoxins. In both cases, C5a induced stronger responses than C3a, and interfering effects were observed when the cells were co-stimulated with C3a and C5a. Parallel experiments with monocytes using the same anaphylatoxin concentrations used with RPE cells revealed the same effects on cytokine secretion, although to a lesser degree (Figure S1 in Supplementary Material).

## Discussion

The main results of the current study are: (1) Human RPE expresses C3aR and C5aR and (2) responds to anaphylatoxin stimulation with a characteristic slow increase in free cytosolic Ca^2+^. (3) The intracellular Ca^2+^ increase in response to C5aR stimulation involves PI3-kinase-Akt signaling with weak contribution of the L-type calcium channel. The molecular signaling pathways leading to a rise in free Ca^2+^ in response to C3aR stimulation remain unclear. (4) In response to anaphylatoxin stimulation, Akt-dependent FoxP3 and FOXO1 phosphorylation is induced, (5) and the intracellular Ca^2+^ increase correlates with increased CREB phosphorylation. (6) Finally, complement gene expression is not altered in response to anaphylatoxin stimulation, but secretion of IL-8 and VEGF-A increases in response to co-application of C3a and C5a. (7) Overall, in both ARPE-19 and monocytes, C3a-elicited responses are weaker compared to those elicited by C5a. (8) In most cases, responses are either driven by C5a (C3a + C5a = C5a) or are smaller than those elicited by C3a alone (C3a + C5a < C3a). In summary, our data suggest that the RPE shares certain characteristics with immune cells (FoxP3 expression, cytokine secretion), confirming its role in the “immunoregulatory” gate (47–49); the RPE thus actively contributes to the establishment of a pro-inflammatory environment in the presence of complement activation. The differential responses to single and co-administration of anaphylatoxins allows for graded responses by the RPE. As C5aR engagement usually dominates and/or reverses C3a-receptor-mediated responses, anaphylatoxin signaling should be tested within the context of the intact complete complement system.

Although a large body of evidence describes a prominent role for chronic local complement activation in AMD, and the presence of anaphylatoxins in pathological structures of the RPE have been described (25), the impact of complement proteins on the RPE has not yet been investigated carefully. To date, research has focused mostly on the effects of the TCC or MAC on cellular readouts (26). Initially, while C3aR and C5aR could not be demonstrated in the RPE by immunohistochemistry (50), gene expression data in ARPE-19 cells (13) as well as our data from primary human RPE cultures and our unpublished microarray analyses on RPE/choroid from C57BL/6J mice (U74Av2, Affymetrix; C3aR, *p* = 0.001; C5aR, *p* = 0.05) suggest that both receptors are expressed. Functionally, we have previously shown that RPE cells do respond to complement activation products by analyzing the ionic mechanisms of complement-evoked Ca^2+^ signals. Using C3- or C5-depleted sera, we found specific contributions of anaphylatoxins to Ca^2+^-signaling (19). Here, we looked in more detail by investigating the anaphylatoxin-evoked rises in intracellular free Ca^2+^ as a second messenger in the absence of other confounding NHS components. Both C3a and C5a increased intracellular Ca^2+^, with the C5a responses being about 2x as those elicited by C3a. The C5a-driven Ca^2+^-signal required the activation of PI3-kinase and Akt, as demonstrated by pharmacological intervention or by direct assessment of either Akt or PI3-kinase phosphorylation. These data are in accordance with other studies that have investigated either C5aR or C3aR activation in immune cells (28). Since the PI3K-isoforms that specifically regulate ion channel activation and thus Ca^2+^ signals have so far not been identified, we used LY294002 as a pan PI3-kinase blocker. It is likely that anaphylatoxin-activated pathways involve different PI3K-isoforms for ion channel regulation and/or for transcription factor activation. However, deciphering these complex interactions would require its own study.

We have previously shown that NHS generated Ca^2+^ elevations that are mainly driven by the activation of voltage-dependent L-type Ca^2+^ channels at the initial peak and a following plateau phase. This goes along with increased phosphorylation of the pore-forming Ca_V1.3_ subunit (15, 17, 19). Furthermore, the activation of L-type channels represents an important prerequisite for the effects observed by sub-lytic MAC on RPE cells, especially their increased secretion of VEGF (15, 17). In comparison, we here show that C3a-evoked Ca^2+^ responses were unaffected by the L-type channel blocker nifedipine, whereas the peak response elicited by C5a was significantly reduced; nifedipine in the presence of both C3a and C5a was not tested. Nevertheless, the phosphorylation of the L-type channel pore-forming subunit Ca_V1.3_ was increased by both anaphylatoxins alone, or in combination. Finally, similar to the stimulation in NHS (12), C3a and C5a together increased VEGF secretion in ARPE-19 cells. Taken together, the majority of readouts triggered by NHS (Ca^2+^-signal and its susceptibility to nifedipine blockage, Ca_V1.3_ phosphorylation, and VEGF secretion) were replicated by co-administration of C3a and C5a and in some instances by C3a or C5a alone. Since C3a or C5a alone did not generate the L-type channel-dependent Ca^2+^-signal like in NHS, other complement cascade components, especially the TCC/MAC, may participate in NHS-dependent Ca^2+^ signaling.

This complexity of the complement system is reflected in the results of our experiments, which revealed interactions between intracellular signaling pathways triggered by C3aR and C5aR. Calcium imaging allowed for the most careful dissection of the anaphylatoxin-evoked signals. Those experiments showed that C3a and C5a each elicited a Ca^2+^ response, but when co-administered, the response was not additive, but rather appeared to be limited in amplitude to the response elicited by C5a alone. When administered sequentially, C5a was found to reduce the already established C3a response, rather than increasing it. Given the above-indicated multitude of involved intracellular signal molecules, this interaction between C3a and C5a signaling might occur on a variety of different levels between ionic mechanisms and kinases. The most obvious mechanism, an acute decrease of C5aR surface expression induced by C3a, was ruled out by analysis of C5aR expression after C3a stimulation in human monocytes (Figure S1 in Supplementary Material).

The activation of CREB, which is known to transduce Ca^2+^ elevations into changes in gene expression (41, 42), suggests that this pathway might play a significant role; however, differential effects between C3a, C5a, and the combined exposure on CREB were not identified. The generation of anaphylatoxin-evoked Ca^2+^ signals in immune cells has been shown to activate the transcription factors FOXO1 and FoxP3 to change the activation status of the cell (27, 28). Similarly, here, we found phosphorylation of FOXO1 and FoxP3 in RPE cells as a result of anaphylatoxin receptor stimulation. FoxP3 phosphorylation showed the same profile as the Ca^2+^ signal; the C3a-driven phosphorylation was smaller in magnitude than that driven by C5a, and when co-administered, the response was again limited in amplitude to the response elicited by C5a alone. This is in contrast to FOXO1 phosphorylation, which showed an additive effect.

These anaphylatoxin-mediated changes in the RPE resulting in changes in ionic composition and activation of transcription factors might lead to profound changes in RPE function. While no changes were observed in gene expression levels of C3, C5, or the two anaphylatoxin receptors after stimulation of C3a or C5a alone, the combined C3a/C5a stimulation reduced C3 expression when compared with control. Similarly, no significant changes were observed for the secretion of IL-8 and VEGF after stimulation with C3a or C5a alone, but the combined C3a/C5a stimulation significantly increased IL-8 and VEGF levels when compared with control. We propose that the combined activation of C3aR and C5aR triggers the reactivity of RPE cells to inflammation, but not when individual receptor systems are activated separately.

A surprising finding in our study is FoxP3 expression and phosphorylation in response to anaphylatoxin stimulation in RPE cells. FoxP3 protein expression has been postulated to be restricted to T lymphocytes (30, 44, 51); to our knowledge, this is the first report that this protein is detected in other cells. While FoxP3 was not detected in native human RPE or in healthy rat eyes, under inflammatory conditions such as during experimental uveitis, FoxP3 protein expression was detected in rat RPE cells. Phosphorylation of FoxP3 in ARPE-19 cells in response to stimulation with anaphylatoxins supports a role for FoxP3 as a reaction to dangerous events. Whether FoxP3 expression in stressed RPE cells allows them to acquire a regulatory cell phenotype to protect the retina from inflammatory insults and to maintain the ocular immune privilege or facilitate the crosstalk with the immune system under potentially dangerous conditions will be investigated in future experiments.

In general, signaling *via* C3aR is reported to trigger regenerative, protective, and anti-inflammatory responses, whereas signaling *via* C5aR triggers immune cell recruitment and inflammation (28). These conclusions are supported by animal studies using application of anaphylatoxins and knock-out mouse models (51–57). Here, we have identified mechanisms that could mediate the different reactions of the RPE in response to C3a, C5a, and the combination of C3a/C5a; in particular, our data suggest that C3aR engagement prevents further C5aR responses, possibly favoring C3a-dependent effects. This mechanism is supported by data analyzing end organ damage in C3aR- and C5aR-deficient mice. C3aR deficiency did not or only marginally reduce end organ damage, whereas the C5aR-deficient mice in some cases even showed augmented regeneration. However, the C3aR/C5aR-double knock-out phenotype did not reproduce C5aR-dominated effects on tissue destruction, but instead showed less severe organ damage than the C3aR single knock-out (51–57).

In summary, we conclude that the RPE behaves in part like an immune cell in its reaction to anaphylatoxin exposure. Thus, we suggest that the special integrative signaling between C3aR and C5aR helps the RPE to precisely adopt its immune-suppressive function. This may lead to a new understanding of the chain of events leading to AMD.

## Author Contributions

CB: planning and conduction of experiments, data analysis, and figure/manuscript preparation. BA, KA, NR, CH, YL, EJ, and MD-M: conduction of experiments. PZ: preparation of manuscript. CS and GW: conduction of experiments, data analysis, and figure/manuscript preparation. BR and OS: planning of experiments, data analysis, and figure/manuscript preparation.

## Conflict of Interest Statement

The authors declare that the research was conducted in the absence of any commercial or financial relationships that could be construed as a potential conflict of interest.

## Supplementary Material

Figure S1 **(A)** Secretion of interleukin-1 beta (IL-1β), interleukin-6 (IL-6), and interleukin-10 (IL-10) was tested in human monocytes stimulated with C3a (330 nM), C5a (48 nM), or both when compared with buffer alone. Level of cytokine induction by C5a was arbitrarily set as 1. Data are expressed as mean values ± SD; *n* = 3; **p* < 0.05, Student’s *t*-test. **(B)** FACS analysis of C5aR surface expression in human monocytes after stimulation with BSA (gray), C3a (3 µM, green), or C5a (0.6 µM, red).

Figure S2 **(A)** Cryosections of rat eye with experimental uveitis induced by immunization as described and double stained for TCR-αβ and -γδ as well as FoxP3 expression. Left side (I, III): FoxP3-Cy3 staining (red), right side (II, IV): TCR-αβ/γδ-FITC staining (green). Upper panel: T cells (arrows) infiltrating the retina (II), but no concomitant FoxP3 staining [I, retinal pigment epithelium (RPE) was destroyed during uveitis]. Lower panel: FoxP3 staining of the RPE (asterisk) after peak disease (III), no more T cell infiltrates visible (IV). **(B)** Control immunofluorescence staining of a Lewis rat (albino) eye section with experimental uveitis: secondary Cy3-labeled donkey anti-rabbit IgG antibody only. RPE is identified by an asterisk. **(C)** FoxP3 antibody-positive control: rat spleen section stained for FoxP3 expression. G = germinal centers surrounded by FoxP3-positive cells.
