# Supplementary figures and images for: Anaphylatoxins Activate Ca2+, Akt/PI3-Kinase, and FOXO1/FoxP3 in the Retinal Pigment Epithelium
Source: Front Immunol. 2017 Jun 15;8:703. doi: 10.3389/fimmu.2017.00703 (PMC5472091; doi:10.3389/fimmu.2017.00703)

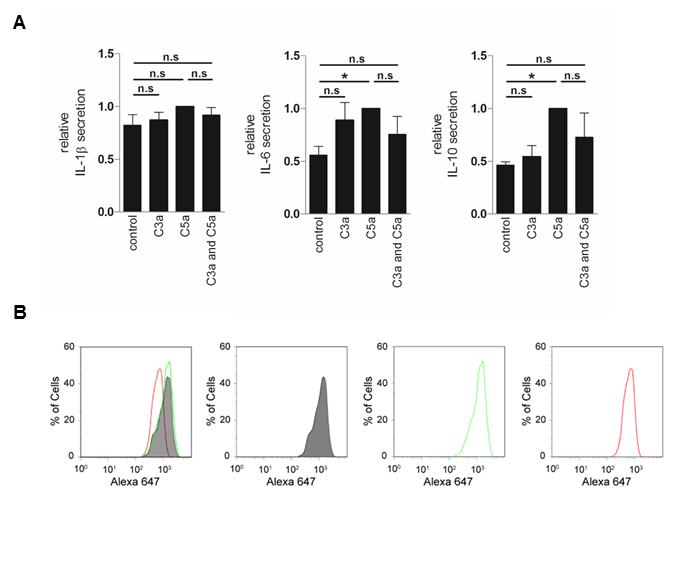

Supplement: Figure S1 — (A) Secretion of interleukin-1 beta (IL-1β), interleukin-6 (IL-6), and interleukin-10 (IL-10) was tested in human monocytes stimulated with C3a (330 nM), C5a (48 nM), or both when compared with buffer alone. Level of cytokine induction by C5a was arbitrarily set as 1. Data are expressed as mean values ± SD; n = 3; *p < 0.05, Student’s t-test. (B) FACS analysis of C5aR surface expression in human monocytes after stimulation with BSA (gray), C3a (3 µM, green), or C5a (0.6 µM, red). [file Image_1.tif]

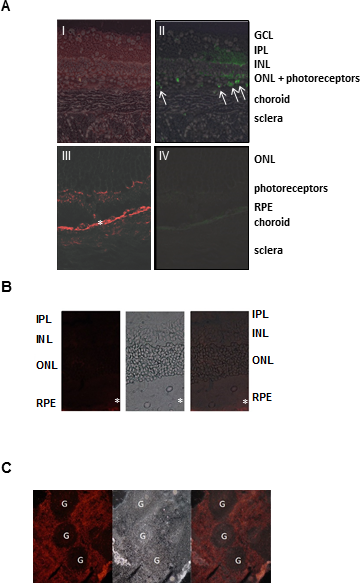

Supplement: Figure S2 — (A) Cryosections of rat eye with experimental uveitis induced by immunization as described and double stained for TCR-αβ and -γδ as well as FoxP3 expression. Left side (I, III): FoxP3-Cy3 staining (red), right side (II, IV): TCR-αβ/γδ-FITC staining (green). Upper panel: T cells (arrows) infiltrating the retina (II), but no concomitant FoxP3 staining [I, retinal pigment epithelium (RPE) was destroyed during uveitis]. Lower panel: FoxP3 staining of the RPE (asterisk) after peak disease (III), no more T cell infiltrates visible (IV). (B) Control immunofluorescence staining of a Lewis rat (albino) eye section with experimental uveitis: secondary Cy3-labeled donkey anti-rabbit IgG antibody only. RPE is identified by an asterisk. (C) FoxP3 antibody-positive control: rat spleen section stained for FoxP3 expression. G = germinal centers surrounded by FoxP3-positive cells. [file Image_2.tif]
